# Coping with Unusual ExperienceS for 12–18 year olds (CUES+): a transdiagnostic randomised controlled trial of the effectiveness of cognitive therapy in reducing distress associated with unusual experiences in adolescent mental health services: study protocol for a randomised controlled trial

**DOI:** 10.1186/s13063-017-2326-4

**Published:** 2017-12-04

**Authors:** Suzanne Jolley, Sophie Browning, Richard Corrigall, Kristin R. Laurens, Colette Hirsch, Karen Bracegirdle, Kimberley Gin, Francesca Muccio, Catherine Stewart, Partha Banerjea, Elizabeth Kuipers, Philippa Garety, Majella Byrne, Juliana Onwumere, Evanthia Achilla, Paul McCrone, Richard Emsley

**Affiliations:** 1 Department of Psychology, King’s College London, Institute of Psychiatry, Psychology and Neuroscience, 16, De Crespigny Park, Denmark Hill, London, SE5 8AF UK; 2 South London and Maudsley NHS Foundation Trust, London, SE5 8AZ UK; 3 School of Psychology, Faculty of Health Sciences, Australian Catholic University, Banyo, QLD 4014 Australia; 4 Department of Forensic and Neurodevelopmental Sciences, King’s College London, Institute of Psychiatry, Psychology and Neuroscience, London, SE5 8AF UK; 5 Research Unit for Schizophrenia Epidemiology, School of Psychiatry, University of New South Wales, Sydney, NSW 2052 Australia; 6 Neuroscience Research Australia, Randwick, NSW 2031 Australia; 7 National Institute for Health Research Mental Health Biomedical Research Centre and Dementia Unit (BRC/U) at the South London and Maudsley NHS Foundation Trust and Institute of Psychiatry, Psychology and Neuroscience, King’s College London, London, SE5 8AZ UK; 8 Department of Health Service and Population Research, King’s Health Economics, King’s College London, Institute of Psychiatry, Psychology and Neuroscience, London, SE5 8AF UK; 9 Centre for Biostatistics, School of Health Sciences, The University of Manchester, Manchester Academic Health Science Centre, Manchester, M13 9PL UK; 10 Manchester Academic Health Science Centre Clinical Trials Unit, Manchester, M13 9PL UK

**Keywords:** Child, psychotic-like experience (PLE), Community mental health, Cognitive behavioural therapy (CBT)

## Abstract

**Background:**

Childhood ‘unusual experiences’ (such as hearing voices that others cannot, or suspicions of being followed) are common, but can become more distressing during adolescence, especially for young people in contact with Child and Adolescent Mental Health Services (CAMHS). Unusual experiences that are distressing or have adverse life impact (UEDs) are associated with a range of current and future emotional, behavioural and mental health difficulties. Recommendations for psychological intervention are based on evidence from adult studies, with some support from small, pilot, child-specific evaluations. Research is needed to ensure that the recommendations suit children as well as adults. The CUES+ study (Coping with Unusual ExperienceS for 12–18 year olds) aims to find out whether cognitive behaviour therapy for UEDs (CBT-UED) is a helpful and cost-effective addition to usual community care for 12–18 year olds presenting to United Kingdom National Health Service Child and Adolescent Mental Health Services in four London boroughs.

**Methods:**

The CUES+ study is a randomised controlled trial comparing CBT-UED plus routine care to routine care alone. CBT-UED comprises up to 16 sessions, including up to 12 individual and up to four family support meetings, each lasting around 45–60 min, delivered weekly. The primary outcome is emotional distress. Secondary outcomes are change in UEDs, risk events (self-harm, attendance at emergency services, other adverse events) and health economic outcomes. Participants will be randomised in a 1:1 ratio after baseline assessment. Randomisation will be stratified by borough and by severity of mental health presentation: ‘severe’ (an identified psychotic or bipolar disorder) or any ‘other’ condition. Outcomes will be assessed by a trained assessor blind to treatment condition at 0, 16 and 24 weeks. Recruitment began in February, 2015 and is ongoing until the end of March, 2017.

**Discussion:**

The CUES+ study will contribute to the currently limited child-specific evidence base for psychological interventions for UEDs occurring in the context of psychosis or any other mental health presentation.

**Trial registration:**

International Standard Randomised Controlled Trials, ID: ISRCTN21802136. Prospectively registered on 12 January 2015. Protocol V3 31 August 2015 with screening amended.

**Electronic supplementary material:**

The online version of this article (doi:10.1186/s13063-017-2326-4) contains supplementary material, which is available to authorized users.

## Background

Psychosis is a disabling and costly mental health condition [1–6] with adverse social and functional outcomes, even following an at-risk presentation [7, 8]. For adults with psychosis, individual and family based cognitive behavioural therapy/interventions (CBTp and FIp, respectively) are recommended by the United Kingdom National Institute for Health and Care Excellence (UK NICE) [5, 6]. On the strength of the adult evidence base, similar recommendations are made for children and young people under the age of 18 years [4, 9]. The guidance extends the offer of treatment to childhood presentations of psychotic-like, or unusual experiences (such as hearing voices that others cannot, or unfounded worries about being followed or deliberately harmed) in the absence of a formal diagnosis of psychosis, or any other condition, when these are accompanied by distress or adverse life impact (UEDs).

Around 15% of young people in the general population experience UEDs, with the rate increasing to around half of young people referred to Child and Adolescent Mental Health Services (CAMHS) with emotional and behavioural problems [10–14]. The occurrence of non-distressing unusual experiences is higher in younger childhood, and frequency generally decreases with age. However, as frequency decreases, the likelihood of associated distress/adverse impact increases [10, 15, 16]. Severity, distress and less effective coping skills predict a persisting trajectory [17]. UEDs have been associated with a range of poor mental health outcomes and intervention is indicated to reduce current distress and disability, with the potential to additionally increase resilience and reduce future mental health risk [12, 13, 18, 19]. However, for younger adolescents (aged under 14 years), recent guidance suggests that the lack of specificity of UEDs as a risk factor for psychosis contraindicates explicitly preventative interventions [20, 21].

Cognitive behavioural models of psychosis assume a continuum of experience, such that the same psychological processes are hypothesised to drive both the persistence and severity of the cognitive, emotional and behavioural difficulties that are characteristic of psychosis, irrespective of whether or not they reach criteria for a diagnosis [22, 23]. Consistent with this, cognitive therapy has been demonstrated to reduce transition to psychosis for young people (usually aged 14 to 35 years) presenting with an at-risk mental state [24]. However, non-transition is still associated with difficulties in health and functioning, and intervening earlier may offset the accumulation of damaging personal, social and economic effects [7, 8, 25].

UK NICE guidance emphasises the need for trials in children, to build a youth-specific evidence base, both for early intervention with UEDs and for working with young people with UEDs in the context of an identified psychotic condition. Emerging evidence suggests that at least some psychological targets of cognitive therapy are common between adult psychosis and childhood UEDs [11, 14, 26–29], supporting the use of similar therapeutic strategies. However, research also suggests that standard protocols are less effective for younger participants [30]; in a recent trial, for younger participants (mean age 16.5 years, range 14 to 30 years), unadapted CBTp performed no better than generic support [31]. We have shown, in pilot and case series work, that adapted, child-specific cognitive behavioural interventions targeting adolescent psychosis or childhood UEDs are feasible, acceptable, safe and potentially helpful [32–34]. This trial will test our child-specific therapy adaptations, which include: shorter duration of sessions and of therapy as needed; greater emphasis on behavioural change; explicit connection with educational, social, familial, and particularly peer, context, and a focus on the developing sense of self and identity. Given the importance of the familial context [35–38], and as the majority of young people are living with an adult carer (usually an extended family member in a parental role), the intervention includes up to four sessions of family work. The role of the family environment in psychosis in adults has been convincingly demonstrated [37]. Early evidence suggests that parents of young people with UEDs, although often unaware of their child’s unusual experiences, are aware of associated emotional and behavioural difficulties [10, 14, 36]. Parents of young people with UEDs experience higher levels of affective disturbance than the general population, and difficulties in their relationship with their child [36]. Associations between parental criticism, social isolation, coping and affective disturbance, are consistent with a cognitive model of caregiving in psychosis [36, 37]. There are suggestions that parental criticism can exacerbate UEDs [38]. Therefore, it is particularly important to offer family support alongside individual interventions for our target population.

### Study aims

We plan to carry out a phase II interventional, randomised controlled trial, to test the clinical and cost-effectiveness of our adapted CBT for childhood UEDs (CBT-UED), as an adjunct to routine care (treatment as usual, TAU), in reducing distress in adolescents (aged 12–18 years) with UEDs in the context of psychosis or any other presentation, in community CAMHS. The CBT-UED intervention plus TAU condition will be compared to TAU alone. Young people allocated to TAU will be offered the CBT-UED intervention after completing the final trial assessment.

The specific research questions to be addressed are:

1. Are clinical outcomes for young people with UEDs improved by the addition of CBT-UED to routine care?
2. Are the effect sizes comparable to those found in the adult academic literature?
3. Is the intervention cost-effective?

## Methods

### Participants and setting

We aim to recruit 120 young people aged between 12 and 18 years, presenting to CAMHS in four London boroughs served by the South London and Maudsley National Health Service Foundation Trust: Southwark, Lambeth, Croydon and Lewisham. The sample will be transdiagnostic, and will include young people with a diagnosed or emergent psychosis, or any other presentation, treated by the clinical team. However, all participants will have unusual experiences in the form of positive psychotic or psychotic-like phenomena, and associated distress/adverse life impact.

Each borough service carries a caseload of around 500 young people, with around 60 new referrals per month per borough and a workforce of around 70 community mental health workers in total. The teams work with young people whose presentation warrants secondary mental health care; the usual criteria are risks of harm to themselves or others. National incidence rates of psychosis may be exceeded in the target services: a team with 40–60 new referrals/month averages three psychosis referrals/month. Population estimates and our recent work indicate that at least an additional 25–50% of referrals will have UEDs, in the absence of a psychosis diagnosis [10–14]. Based on approaching all new referrals, and assuming a consent rate of 50%, we estimated a recruitment rate of six young people/month.

#### Screening protocol

In order to participate in the CUES+ study, young people need to present with unusual experiences with accompanying distress and/or adverse life impact. Following our earlier pilot work with the target services [34], borough teams routinely administer a measure of unusual experiences (the Unusual Experiences Questionnaire, UEQ) [10, 14, 39, 40] with their standard assessment battery. The UEQ has been shown to be a reliable and valid measure of UEs for young people [12]. Respondents rate each of nine UEs on a 3-point Conviction scale: 0 (not true); 1 (somewhat true); 2 (certainly true); endorsed UEs are rated for Frequency over the past 2 weeks: 0 (not at all); 1 (only once); 2 (2–4 times); 3 (5 + times); Distress (‘How much has it upset you?’) and Adverse Impact (‘How much has it made things hard at home or school?’), both rated: 0 (not at all); 1 (only a little); 2 (quite a lot); 3 (a great deal). Item totals (ratings across dimensions of conviction, frequency, distress, impact, range 0–11), are summed to create a UE-severity score, and, by selecting only those items where distress or impact is rated > 0, a UED-severity score (0–99, higher scores indicate greater severity). We have found this screening procedure to be both feasible and acceptable to young people and their families [34]. Screening is important as UEDs are not otherwise routinely assessed in community CAMHS, and, although parents and other involved agencies may be aware of distress, or behavioural problems, children tend not to report their UEDs unless directly asked [10, 14]. Young people also routinely complete a measure of childhood psychopathology (the Strengths and Difficulties Questionnaire, SDQ) [41, 42]. We initially screened for young people rating any UE > 0 on UEQ conviction, who also had a score in the clinical range (≥7) of the Emotional Problems subscale of the SDQ (SDQ-E) to indicate current distress. However, early recruitment figures (after 6 months of recruitment) showed that 80% of young people endorsing unusual experiences but not meeting the SDQ-E distress criterion were reporting distress and/or adverse impact associated with the unusual experience, and expressing interest in participating in the study. With the agreement of our funders, the clinical service, our Independent Trial Steering Committee and our Research Ethics Committee (REC), we therefore amended the criteria to also include endorsing a UE, with self-rated distress or adverse impact ≥ 0, with no requirement to meet the clinical criterion of the SDQ-E. This was agreed sufficiently close in time to the trial start (December, 2015) for young people who had been excluded at the screening stage to be recontacted regarding participation.

#### Inclusion and exclusion criteria

Inclusion criteria are: presenting to local CAMHS; current unusual experience with associated distress and/or adverse impact; aged 12–18 years; intending to be available for the next 6 months in order to complete participation in the study; sufficient English language ability for young people, and parents as relevant, to be able to give informed consent (or assent with parental consent), complete assessment measures and participate in therapy, with interpreter support as appropriate. Exclusion criteria are: having a known learning disability (Intelligence Quotient, IQ < 70, confirmed by the treating team); a UED occurring only secondary to a known neurological condition (e.g. epilepsy or brain injury); limited to states of acute intoxication/withdrawal in the context of substance misuse.

### Study design

**Fig. 1:**
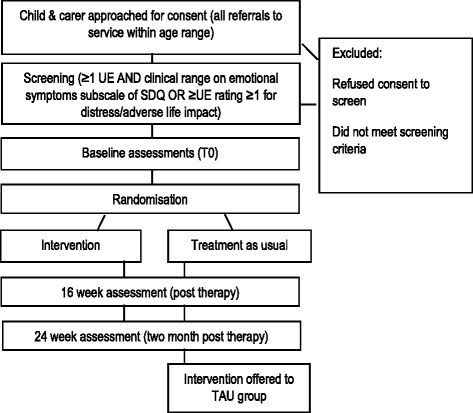
Coping with Unusual ExperienceS for 12–18 year olds (CUES+) study design. Key: *UE* Unusual experience, *SDQ* Strengths and Difficulties Questionnaire [41, 42], *TAU* Treatment as usual (routine care)

**Fig. 2:**
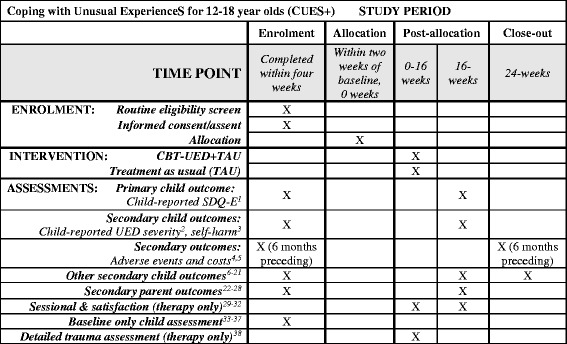
Coping with Unusual ExperienceS for 12–18 year olds (CUES+): schedule of enrolment, interventions and assessments. Key: *UED* unusual experience with distress, *CBT* cognitive behavioural therapy, ^1^Strengths and Difficulties Questionnaire (SDQ), Emotional Problems subscale [41, 42]. ^2^Unusual Experiences Questionnaire (UEQ) – severity score [10, 14, 39]; ^3^from the Development and Wellbeing Scales (DAWBA) [45]. ^4^Child adaptation of the Clinical Service Receipt Inventory [51]; ^5^EuroQol Health Questionnaire, youth version (EQ5D-Y) [52–54]. ^6,7^Parent- and child-reported full SDQ and UEQ (including UEQ appraisals [68, 72]; ^8,9^researcher-reported functioning (Child Global Assessment Scale, Health of the Nation Outcome Scales for Children and Adolescents) [86–88];^10–12^child- and parent-reported mood and behaviour (Revised Child Anxiety and Depression Scales [55]; Elevated Mood Scales [56–58]; Me and My School, behavioural problems subscale [59]; ^13–17^child-reported trauma sequelae/emotion regulation (Difficulties in Emotion Regulation Scale [60]; Adolescent Dissociative Experiences Scale [61]; Child Revised Impact of Events Scale-13 [62]; DAWBA eating disorder screen [45]; Maudsley Addiction Profile [63]); ^18^Time budget measure of activities and peer relationships [64–66]; ^19^Beliefs about problems [69–72]; ^20^Brief Core Schema Scale [27, 68]; ^21^Jumping to conclusions reasoning task [26, 73]; ^22–24^Parent caregiving experience and appraisals (Five Minute Speech Sample [74]; Brief Illness Perception Questionnaire [36, 69]; Experience of Caregiving Inventory [78]; ^25–28^parent distress/wellbeing and coping (Patient Health Questionnaire, Generalised Anxiety Disorder, Short Warwick-Edinburgh Mental Well-Being Scale, Brief COPE Scale with Confidante Question [36, 75–78]. ^29–32^Short CHOICE with goals [84]; Session and Outcome Rating Scales [79–82]; service satisfaction [83]; ^33^demographics (age, gender, ethnicity, parent-reported developmental delay); ^34^life events, bullying and brief trauma screen [69.70,45]; ^35-37^Wechsler Intelligence Scales for Children – Fourth Edition (WISC-IV), Individual Attainment Test II (WIAT-II), Abbreviated Scale of Intelligence II (WASI-II) [48–50]. ^38^Children’s Post-Traumatic Cognitions Inventory [44]

CUES+ is a parallel-group RCT with random allocation to one of two arms, comparing our active intervention (CBT-UED + TAU) to routine care alone (TAU). Treatment as usual (TAU) will be delivered without interference in both conditions and includes care coordination, practical and emotional support for the young person and their family, and medication as appropriate. We will record what is delivered as routine care. Assessments will take place at baseline (0 weeks), 16 weeks (post therapy) and 24 weeks (2 months post therapy). After 24 weeks, TAU participants will be offered the intervention. Trained research workers will complete assessments with parents and young people. Baseline assessments will be carried out prior to randomisation; 16- and 24-week assessments will be arranged by the trial research worker, but carried out by a different assessor who will be blind to treatment allocation. Service and economic measures will be completed for the 6-month periods before and after baseline. The study design is illustrated in Figs. 1 and 2 (Fig. 2 shows a modified version of the Standard Protocol Items: Recommendations for Interventional Trials (SPIRIT) Figure for the trial).

### Procedure

#### Recruitment

We will recruit directly from CAMHS community mental health teams, starting in one borough, and extending to the other boroughs as required to maintain the target recruitment rate. Young people presenting with UEDs (according to the screening measure) will be invited by their team to find out more about the study, and, if agreeing, will be contacted by the CUES+ study researcher. The usual consent procedure for participants aged under 16 years will be to secure parental consent and child assent; and for those 16 years and over, to seek the young person’s consent, and parental consent if the child agrees to familial participation. This will be guided by the clinical team’s assessment of the young person’s capacity and other clinical issues impacting on consent (e.g. whether Gillick competences [43] are met, managing parental conflict and split care arrangements). Young people reporting UEDs (and their families where appropriate) will be offered the Information Sheet and Consent/Assent Forms, with a follow-up call from the research team within 2 to 4 weeks. Telephone conversations and meetings will take place as needed to discuss the study; at least 24 h will elapse between receipt of the study information and consent being sought for participation. Consenting young people and their families will be offered a baseline assessment and will be randomised only following completion of this.

#### Ethical approval

The study has been reviewed and given a favourable opinion by the London Hampstead National Research Ethics Service Committee (reference: 14/LO/1970). Approval to recruit for the study in CAMHS in the four boroughs of Southwark, Lambeth, Croydon and Lewisham was granted centrally by the Joint Research and Development Office of the South London and Maudsley NHS Foundation Trust and the Institute of Psychiatry, Psychology and Neuroscience (reference R&D2015/003).

#### Intervention

The therapy will comprise up to 16 sessions, delivered over 16 weeks, including up to 12 sessions of individual CBT, adapted for adolescents, and up to four family support sessions. The intervention will be delivered in addition to routine care (specialist care coordination, practical and emotional support for the young person and their family, and medication as appropriate), and will be compared to routine care alone. After the 24-week assessment, adolescents in the routine care condition will be offered the intervention.

Intervention will not ordinarily exceed a total of 16 sessions (notwithstanding requirements to extend therapy to ensure safety and wellbeing), but may exceed 16 weeks of delivery, as needed to accommodate, for example, holidays and examination periods. The intervention content has been developed from our earlier inpatient protocols for individual and family work with young people with psychosis [32], and adapted for young people with UEDs in the general population [33] and in services for children with emotional and behavioural problems [34]. Pilot work has demonstrated feasibility, acceptability and potential helpfulness of the intervention [32, 33]. Individual work focusses on developing a collaborative understanding of UEDs, together with skills in affect regulation, managing negative automatic thoughts, behavioural tests, dealing with social difficulties and adverse life events, recognising and compensating for cognitive biases, and a section on taking the work forward and preventing future difficulties. Where there has been trauma, therapists complete pre- and post-therapy measures of event appraisals and post-traumatic cognitions to aid therapy [44]. Therapy is tailored to take account of the developmental stage and presenting issues of the child/young person, with an emphasis on identity formation, understanding of the self in relation to the experience of psychosis/UEDs, social inclusion and self-esteem. Therapy materials are designed to be fun, interactive and engaging. Family support is crucial when working with young people, and has been requested by parents consulting on our studies. Family work comprises recognition and understanding of the child’s difficulties, sharing the intervention plan, and troubleshooting any key familial difficulties.

#### Therapists

We will train and supervise CAMHS community therapists to deliver the intervention. Therapists will contribute a full day or half a day per week, depending on the agreement with their service, to see two to four trial cases. The combined caseload across all therapists, given the study recruitment targets, will be a maximum of 24 at any one time, allowing 60 young people to be seen over a year. We will stagger the input of the therapists to accommodate the growing caseload as the study progresses.

Therapists will be trained to competence in delivery of the manualised intervention and closely supervised by the trial coordinator/supervisor. Therapy adherence will be monitored by supervisors on an ongoing basis through audio-recorded therapy sessions (for which we will request service user consent). Adherence to the manual will be checked by internal raters who are not directly involved in the care of the participant (i.e. not therapist or supervisor), using checklists of manual content incorporating general cognitive therapy skills, specific skills in working with UEDs, and appropriate tailoring to the developmental stage and presentation of each young person and family [32, 33]. An independent expert will rate 10% of internally rated sessions: we will consider 90% rated as adherent to be acceptable. For the duration of the trial, we will ensure that trained therapists do not provide care coordination for young people who are allocated to routine care. In each team, there is an early-intervention-in-psychosis liaison worker, who takes responsibility, irrespective of the trial, for ensuring that routine care meets the needs of young people presenting with psychosis.

### Measures

#### Outcomes and timeline

Following informed consent from each participant, all outcomes will be assessed by a trained study researcher. Demographic/clinical characteristics (age, gender, ethnicity, parent-reported developmental delays, family circumstances, diagnoses), brief cognitive functioning (word reading, working memory and general intelligence) and history of adverse life events and bullying will be assessed at baseline [45–50]. Outcomes will be assessed at baseline (0 weeks) then at 16 weeks and 24 weeks post randomisation, irrespective of treatment duration. Assessments will not be completed more than 2 weeks after the planned assessment time point. Outcomes and all measures are listed in Table 1 and will be completed according to the schedule in Fig. 2.

**Table 1:** Coping with Unusual ExperienceS for 12–18 year olds (CUES+): list of measures

Measure	Completed	
	By	At
Primary outcome		
1. Strengths and Difficulties Questionnaire-Emotional Problems (SDQ-E) [41, 42]	1	0,1
Main secondary outcomes		
2. Unusual Experiences Questionnaire – severity score (UEQ) [10, 14, 39]	1	0,1
3. Self-harm (Development and Wellbeing Scales, DAWBA) [45]	1	0,1
4. Child Clinical Service Receipt Inventory (CSRI) [51]	2,3	− 1,2
5. EuroQol Health Questionnaire, youth (EQ5D-Y) [52–54]	1,2	0,2
Other secondary child outcomes		
6. Strengths and Difficulties Questionnaire (SDQ) [41, 42]	1,2	0,1,2
7. Unusual Experiences Questionnaire (UEQ) [10, 14, 39]	1,2	0,1,2
8. Child Global Assessment Scale (C-GAS) [86]	3	0,1,2
9. Health of the Nation Outcome Scales for Children and Adolescents (HoNOSCA) [87, 88]	3	0,1,2
10. Revised Child Anxiety and Depression Scales (RCADS) [55]	1,2	0,1,2
11. Elevated Mood Scales [56–58]	1,2	0,1,2
12. Me and My School, behavioural problems (M&MS) [59]	1,2	0,1,2
13. Difficulties in Emotion Regulation Scale (DERS) [60]	1	0,1,2
14. Adolescent Dissociative Experiences Scale (A-DES) [61]	1	0,1,2
15. Child Revised Impact of Events Scale (CRIES-13) [62]	1	0,1,2
16. Eating disorder screen (DAWBA) [45]	1	0,1,2
17. Maudsley Addictions Profile (MAP) [63]	1	0,1,2
18. Time budget of activities and peer relationships [64–66]	1	0,1,2
19. Beliefs about problems (BAP) [69–72]	1	0,1,2
20. Brief Core Schema Scale [27, 68]	1	0,1,2
21. Jumping to conclusions [26, 73]	1	0,1,2
Secondary parent outcomes		
22. Five Minute Speech Sample (FMSS) [74]	2	0,1
23. Brief Illness Perception Questionnaire (BIPQ) [36, 69]	2	0,1
24. Parental Experience of Caregiving Inventory (ECI) [36, 78]	2	0,1
25. Depression: Patient Health Questionnaire (PHQ-9) [75]	2	0,1
26. Generalised Anxiety Disorder (GAD-7) [76]	2	0,1
27. Warwick-Edinburgh Mental Well-Being Scale (WEMWBS) [77]	2	0,1
28. The Brief COPE Scale with Confidante Question [36, 78]	2	0,1
Sessional and satisfaction (therapy only)		
29. Short CHOICE with goals [84]	1,2	3
30 and 31. Session and outcome scales (SRS & ORS) [79–82]	1,2	3
32. Service feedback scales [83]	1,2	2
Baseline only measures		
33. Demographics and developmental history	1–3	0
32. Adverse Life Events, bullying and brief trauma screen [45–47]	1	0
33–35. Word reading, digit span, vocabulary, matrix reasoning [48–50].	1,3	0
Detailed trauma assessment (therapy only)		
36. Children’s Post-Traumatic Cognitions Inventory [44]	1,3	4

Key: Completed by: 1 = child; 2 = parent; 3 = researcher/therapist. Completed at: − 1 = 6 months preceding baseline; 0 = 0 weeks, baseline; 1 = 16 weeks; 2 = 24 weeks; 3 = sessionally; 4 = early in therapy

#### Primary outcome

The primary outcome will be distress at 16 weeks, assessed using the SDQ-E, child-reported [41]. The SDQ is well-validated and routinely used in local services for youth up to 19 years [41, 42].

#### Secondary outcomes

Secondary outcomes will be child-reported UED severity at 16 weeks, assessed using the self-report UEQ [10, 14, 39]. Self-reported self-harm [45] and adverse events including attendance at accident and emergency services, and economic costs, including quality-adjusted life years (QALYs) [51–54] will also be assessed.

#### Other clinical outcomes

We will also measure the cognitive, social, emotional and behavioural therapy targets hypothesised to maintain UEDs. These will be assessed at 0, 16 and 24 weeks. Areas assessed include child self-reported mood and behaviour problems (anxiety, depression, elevated mood, anger, hyperactivity, conduct disorder and peer problems) [41, 42, 55–59]; child-reported trauma sequelae, emotional regulation, substance misuse and eating disorders [45, 60–63]; activity levels, social support and sleep pattern [64–66], appraisals of the self and others, UEDs and the presenting problem [27, 28, 67–72]; and reasoning style [73]. Parent/carer measures will be completed at 0 and 16 weeks, and will include the 5-min speech sample to assess family relationships [74], and measures of parental affect, wellbeing, caregiving experience, and coping [75–79], as well as parental assessments of child mood and behaviour difficulties and UEDs [10, 14, 41, 42, 55–59]. Sessional ratings will be used to measure the therapeutic relationship, therapy progress and overall satisfaction [80–85]. The Child Global Assessment Scale [86] and Health of the Nation Outcome Scale for Children and Adolescents [87, 88] will be completed at the 0-, 16- and 24-week time points to assess general childhood psychopathology and impact on functioning.

#### Economic outcomes

The main outcomes of interest will be unit changes in distress levels measured by the SDQ-E (the primary outcome of the RCT) [41, 42], and the QALY gain as measured with the EQ-5D-Y questionnaire at baseline (0 weeks) and 24-week time points. The EQ-5D-Y is a child version of the EQ-5D, which measures health-related quality of life (HRQoL) in young populations using an age-appropriate adjustment to the wording of the original questionnaire [52–54]. The EQ-5D-Y consists of five domains (mobility, self-care, usual activities (e.g. work, study, housework, family or leisure activities), pain/discomfort and anxiety/depression) and each is rated 1 (no problems), 2 (some problems), or 3 (a lot of problems) [54]. UK values will be applied to the distinct health states derived from the EQ-5D-Y to estimate the utility value for each participant at each time point, and the area-under-the-curve methods will be used to calculate the QALYs [53].

#### Service use and costs

Economic analysis will be conducted from the perspective of the national health care system and personal and social services (NHS and PSS), but a wider perspective including costs borne by the participants and their families will also be considered. Potential costs will include lost education for children and lost time from work for parents. The costs of lost education will be estimated using a notional figure for the value of days in education. Intervention costs will include the time spent by CAMHS community therapists to deliver the intervention, including training and supervision costs. The Client Service Receipt Inventory (CSRI) will be adapted and administered to record participants’ use of health and social services for the 6-month periods before and after baseline, respectively [51]. The CSRI has been developed by members of King’s Health Economics and has been widely used in mental and physical health economic evaluations. The data collected through the CSRI will be used to calculate average service costs and total costs of care. All unit costs will be derived using routine data sources, such as the NHS reference costs [89] and the Unit Costs of Health and Social Care [90], as well as study-specific estimates where appropriate. Medication use, such as name/type of drug, dosage levels and frequencies, will be recorded and costs will be calculated based on prices from the *British National Formulary for Children* [91] and the Prescription Cost Analysis [92]. From these, a mean cost per intervention and a mean cost taking into account participant and carer costs will be estimated.

### Sample size

We will recruit 120 participants, randomly allocating 60 per group. From previous experience, we will allow for a conservative loss-to-follow-up of approximately 33% (85% follow-up achieved in an earlier pilot study [34]. With 45 successfully followed up in each group, we would have 80% power to detect between group differences for the smallest achieved effect sizes from previous studies of cognitive therapy with young people (0.6 SD) using a two-group *t* test with a 0.05 two-sided significance level. We will have 90% power to detect effect sizes of 0.7 SD and above. Our pilot work shows between group effect sizes of 0.6 in inpatients [32]. In practice, the power will be increased by using a mixed (random)-effects model allowing for baseline covariates including distress (rather than a simple *t* test) to gain precision in the effect estimates, but this increase is likely to be counteracted by allowing for modest between-therapist variation.

### Randomisation

Randomisation will be carried out after consent to participate in the trial has been given and the baseline assessment has been completed. Participants will be randomised through an independent web-based service provided by the UKCRC-registered King’s Clinical Trials Unit (Reg. No. 053). The randomisation procedure will employ random permuted blocks of random size, which will maintain pre-randomisation allocation concealment. We will stratify the randomisation lists by a Severe Mental Illness (i.e. psychotic illness)/Other Mental Illness factor, since this is an important prognostic factor. We will also stratify by borough for logistical reasons, so that treatment cases are equitably allocated across therapists.

### Blinding procedure

We will not be able to blind participants to treatment group. Similarly, the therapists cannot be blind to allocation as they will deliver the intervention. However, the research workers completing outcome assessments will be blinded to treatment allocation. Should they be accidentally unblinded during the assessment, we will record which outcomes were completed blind and allocate a new assessor for any subsequent assessments. Post-randomisation assessors will work separately from the research and clinical teams to minimise the likelihood of unblinding. We will ask research workers to guess the allocation group for each participant at each assessment as a test of the success of our efforts to maintain blindness. We will report any instances of unblinding in subsequent publications. The end of the trial will be defined as the last follow-up assessment at 24 weeks. Routine-care participants may continue to receive therapy beyond this point, and we will continue to collect sessional measures, but this will be to inform therapy development, implementation and training, rather than as part of the outcomes of the study. Analyses will be completed blind to allocation.

### Trial monitoring and oversight

We have established an Independent Trial Steering Committee (ITSC), comprising an independent experienced trialist as chair, a CAMHS researcher and clinician who are not involved in the trial, the trial statistician and two independent CAMHS carers or service users. Sponsor and funder representatives will be invited as required. We planned for the ITSC to convene annually at 6, 18 and 30 months; meetings have taken place at 9, 15, 21 and 27 months, with a final meeting planned for 35 months. The ITSC will oversee the progress of the trial and will review any proposed protocol changes. Any changes agreed by the ITSC will then be put to all relevant regulatory bodies including the REC, the sponsors and the funder. Approved changes will be updated in the protocol and trial registry.

### Data monitoring

Based on pilot studies, we do not anticipate risks to participant safety as a direct result of the study and will not, therefore, be conducting any interim data analysis and will not convene a separate Data Monitoring Committee. The trial may be prematurely discontinued by the sponsor, the chief investigator or the REC on the basis of new safety information or for other reasons given by the Ethics Committee or Trial Steering Committee.

If the trial is prematurely discontinued, active participants will be informed and no further participant data will be collected. Arrangements will be made directly with the local clinical service to ensure that the safety and wellbeing of the young people and their families is not compromised by this process.

### Data management

We will use paper assessment packs and enter data into electronic databases. Patient data will be pseudonymised for the duration of the study and fully anonymised after the retention period specified in institutional policies (currently, 12 years) has passed. Fully identifiable personal details will be kept on paper in a locked filing cabinet in a locked or occupied office; on secure NHS computers; and, encrypted, on password-protected computers in the university. Pseudonymised data will be stored on personal laptop computers, using recommended secure encryption methods. All trial data will be stored in line with the Data Protection Act [93].

Separate databases will be used for: (1) baseline demographics, (2) repeated clinical measures (separate database for each time point), (3) child and parent measures, (4) allocation, (5) sessional therapy measures, (6) feedback measures and (7) therapy delivery and adherence. The allocation database will be accessible only to the lead research worker (who will not conduct post-baseline assessments) and the trial statistician until the study is completed. Outcome assessments will be carried out by researchers who do not have access to therapy or feedback data. Data will be checked and cleaned against original paper copies and a final database returned to the statistician, who will combine with allocation data for analysis.

### Safety monitoring and adverse event reporting

We will monitor adverse events for all participants by logging any reported by the participant or their network, or the clinical or research team. We will also check the medical record for unreported adverse events at each time point (for the previous 6 months at baseline; since the last assessment at 16 and 24 weeks). Events will be rated for seriousness according to the impact on the participant’s day-to-day life. The event will be considered to be related to the trial if, in the view of the participant, a member of their network, the clinical team or the research team, it is reported to be related. The primary concern in any report will be to work with the clinical team and other emergency services as appropriate to ensure the participant’s safety. Serious adverse events that are related to the trial will be discussed with the ITSC and reported to the trial sponsor and the REC within 15 days.

### Statistical analysis

In accordance with Consolidated Standards of Reporting Trials (CONSORT) [94] principles, we will report all participant flow in the study. Descriptive statistics will be used to summarise assessments of feasibility and acceptability in terms of recruitment, dropout and completeness of therapy. The main efficacy analysis will be via intention-to-treat with data from all participants included in the analysis including those who do not complete therapy. When completion of the full battery of outcome measures is not possible, participants will be offered the opportunity to complete the primary outcome measure only, and as many of the main secondary outcome measures as are tolerated, in person, by telephone, or by email. Every effort will be made to follow up all participants in both arms for research assessments, and the analysis will use, where appropriate, statistical techniques for handling missing data, determined by the extent and distribution of missing data, and any identified demographic or baseline clinical predictors of missing data (age, gender, ethnicity, SDQ-E, UED severity, Severe/Other Mental Illness, borough). The primary hypothesis will be analysed using a linear mixed model allowing for the baseline measurement of SDQ-E and treatment assignment as fixed effects, with SDQ-E at 4 months as the dependent variable. Therapist effects will be modelled by including a random effect for each therapist in the therapy arm, with the control-arm participants considered as being in individual clusters of size 1. The use of a mixed (random)-effect models will allow for estimation of the intra-cluster correlation coefficient, a measure of the proportion of variance in outcome because of therapist effects, which can be used in future applications; no estimate of this is currently available. Secondary outcome measures (excluding economic outcomes) will be analysed using the same approach.

### Health economic analysis

Health economic analysis will be carried out to compare the service use, costs and cost-effectiveness of cognitive behavioural therapy (CBT-UED) as adjunct to the TAU intervention in young people (aged 12–18 years) with UEDs.

Economic analyses will include a cost-effectiveness and a cost-utility analysis with the respective outcomes being; the cost per one unit change in the SDQ-E scores and the cost per QALY gained. Analyses will be carried out primarily on an intention-to-treat basis although other exploratory analyses, such as per-protocol, may also be considered. Data will be analysed at the end of the study; there are no planned interim analyses. Multiple regression methods will be applied to estimate mean differences in costs and effects, using baseline and follow-up data as the dependent variables and the group identifier as an independent variable. Data with missing observations due to loss to follow-up will be examined to determine both its extent and whether it is missing at random or is informative. If data are missing to a sufficient extent, the use of appropriate multiple imputation techniques will be considered.

#### Bootstrap analysis

To account for the likely skewed distribution of cost data, the non-parametric bootstrap method will be used to make cost comparisons between the two groups [95]. Bootstrapping involves repeatedly estimating the incremental cost-effectiveness ratio to account for the uncertainty surrounding the estimates of costs and effects. Likewise, using the net benefit approach, estimates of the proportion of iterations in which the intervention of interest has the maximum expected net benefit (NB), or equivalently, a positive incremental NB will be determined for a range of willingness-to-pay thresholds. The estimates will be produced by repeatedly sampling with replacement from the existing trial population [96]. The results of the bootstrap analyses will be plotted on cost-effectiveness planes (CEPs) and will be used to estimate cost-effectiveness acceptability curves (CEACs), which show the probability of the intervention to be cost-effective subject to a range of thresholds that society would be willing to pay for a unit improvement in the health outcome (e.g. QALYs) [97]. However, using change in distress levels (SDQ scores) as a measure of health status makes a meaningful interpretation of the CEACs difficult, as acceptance thresholds do not apply. In this case, thresholds at which the intervention has, e.g. above 60% likelihood of being cost-effective, will be explored.

#### Sensitivity analysis

Both cost-effectiveness and cost-utility analyses will include deterministic sensitivity analyses around the intervention costs, the costs from lost work (for parents) and lost education (for children) by varying the initial figures within plausible ranges. This will help increase the level of confidence about potentially key drivers of the analyses and will provide insights into the systematic inclusion of such costs in the evaluation of interventions targeted to children.

## Discussion

We have adhered to SPIRIT (Standard Protocol Items: Recommendations for Interventional Trials) [98, 99] guidance in devising and reporting our protocol (Fig. 2; SPIRIT Checklist with Information Sheets and Consent Forms for young person and parent participants are included as Additional files 1 and 2). The trial is funded until the end of September, 2017, and results will be available during 2018. If the intervention shows effects, we will have demonstrated both the feasibility and the usefulness of training CAMHS clinicians to provide specialised interventions for young people with UEDs. The study will be the first youth-specific trial of CBTp, and, should the therapy be successful, will support implementation of government recommendations in CAMHS, as well as informing therapist training models, and future clinical guidance. Economic evidence will potentially inform commissioning, service provision and policy, including workforce development. Findings will be limited by the size of the study, it being the first of its kind, and its location in a single, specialist organisation. Replication on a wider scale, across multiple sites, will be required, We plan to disseminate findings via local academic and clinical networks, through conference presentation and publication. Authorship will be restricted to those making a substantial contribution to the specific publication. We will continue to implement the work locally. and apply for funding for a larger, multisite study to investigate the potential value of implementation across settings.

### Status

Participants began to enter the trial in February 2015. The first participant was randomised on 4 March 2015; 111 participants (of a target of 120) have been randomised to date. Recruitment will continue until 31 March 2017. Final primary outcome data at 16 weeks will be collected by the end of July 2017.

## Additional files

Additional file 1: Coping with unusual experiences for 12–18 year olds (CUES+): SPIRIT Checklist. (DOC 122 kb)

Additional file 2: Coping with unusual experiences for 12–18 year olds (CUES+): Participant Information Sheets and Consent/Assent Forms. (DOC 4408 kb)

## Abbreviations

CAMHS: Child and Adolescent Mental Health Services
CBTp: Cognitive behavioural therapy (for psychosis)
CUES+: Coping with Unusual ExperienceS for Children aged 12–18 years
FIp: Cognitive behavioural family intervention (for psychosis)
NICE: National Institute for Health and Care Excellence
QALY: Quality-adjusted life year
TAU: Treatment as usual
UE(D): Unusual experience (with distress)
