# Supplementary material for: Coping with Unusual ExperienceS for 12–18 year olds (CUES+): a transdiagnostic randomised controlled trial of the effectiveness of cognitive therapy in reducing distress associated with unusual experiences in adolescent mental health services: study protocol for a randomised controlled trial
Source: Trials. 2017 Dec 4;18:586. doi: 10.1186/s13063-017-2326-4 (PMC5716372; doi:10.1186/s13063-017-2326-4)
Supplement: Supplementary file 2 — Coping with unusual experiences for 12–18 year olds (CUES+): Participant Information Sheets and Consent/Assent Forms. (DOC 4408 kb) [file 13063_2017_2326_MOESM2_ESM.doc]

Appendix 1b: Coping with unusual experiences for 12-18 year olds (CUEs+): Participants information sheets and consent/assent forms.
